# Multimodal Endovascular Treatment of Post-Dissection Thoracoabdominal Aneurysm Using Adjunctive Advanced Endovascular Techniques Combined to Branched Repair: Case Report

**DOI:** 10.3390/reports9020155

**Published:** 2026-05-19

**Authors:** Pietro Dioni, Francesco Colamaria, Alessandro Grandi, Gabriele Piffaretti, Stefano Bonardelli, Luca Bertoglio

**Affiliations:** 1 Division of Vascular Surgery, Department of Clinical and Experimental Sciences, School of Medicine, University of Brescia, ASST Spedali Civili of Brescia, 25123 Brescia, Italy; f.colamaria@unibs.it (F.C.); a.grandi004@unibs.it (A.G.); stefano.bonardelli@unibs.it (S.B.); luca.bertoglio@unibs.it (L.B.); 2 Vascular Surgery, Department of Medicine and Surgery, Circolo University Teaching Hospital, School of Medicine, University of Insubria, 21100 Varese, Italy; gabriele.piffaretti@uninsubria.it

**Keywords:** aortic dissection, endovascular procedures, spinal cord ischemia, embolization

## Abstract

**Background and Clinical Significance**: Treatment options for chronic type B aortic dissections (TBADs) remain a topic of ongoing debate. Patients with post-dissection thoracoabdominal aortic aneurysms (PD-TAAAs) are typically younger than those with degenerative TAAAs, and their aortas undergo continuous remodeling over their lifetime. Fenestrated/branched endovascular aortic repair (F/B-EVAR) has shown promising results, but it can be challenged by the presence of a narrow true lumen, which hinders navigation and deployment of bridging components. Moreover, the presence of patent segmental arteries originating from the false lumen may prevent aneurysm shrinkage due to persistent flow, which may also result in insufficient spinal cord protection strategies and an increased risk of spinal cord ischemia. Consequently, multiple endovascular interventions are often necessary to address the persistent anatomical changes in these patients. **Case Presentation**: We present the case of a patient affected by a post-dissecting TAAA who underwent multiple open and endovascular treatment attempts. The presence of prior multiple laparotomies discouraged a new open surgical repair, while the hypertrophic segmental arteries and the presence of a narrow true lumen made standard F/B-EVAR unfeasible. The patient was successfully treated using a combination of different adjunctive advanced endovascular techniques, including minimally invasive segmental artery coil embolization (MiSACE) as a spinal cord preconditioning strategy and prevention of type II endoleak. Moreover, transcatheter electrosurgical septotomy (TES) was used to create a single aortic channel in the presence of a narrow true lumen, which allowed the deployment of a multifeatured, custom-made branched endograft. **Conclusions**: Endovascular repair of post-dissection TAAAs requires a thorough understanding of advanced endovascular adjuncts, which are often combined to overcome the complex anatomical challenges inherent to this disease. Although encouraging results have been reported, both segmental artery embolization for the indications described here and TES warrant further evaluation in prospective multicenter studies to confirm their safety and efficacy.

## 1. Introduction and Clinical Significance

Patients affected by TBAD are often younger and diagnosed with connective tissue disorders (CTDs) more frequently than those with degenerative TAAAs [1]. While open surgery offers acceptable outcomes to those patients in specialized centers [2], advancements in endovascular techniques and major complications after open procedures are shifting preferences toward endovascular options, now mentioned as a possible treatment modality in the 2024 European Society of Cardiology Guidelines [3]. Nevertheless, F/B-EVAR for PD-TAAAs remains challenged by the presence of a narrow true lumen, which hinders catheter navigation and deployment of stentgraft components, two aspects that introduce durability issues to endovascular repair. Moreover, spinal cord ischemia prevention strategies such as the multistage approach, with gradual aneurysmal sac thrombosis, are often unfeasible in those patients, since arteries arising from the false lumen remain perfused until the coverage of the more distal entry tear. Consequently, both staging and complete exclusion of the aneurysm are challenged by the persistent perfusion of the false lumen. Emerging techniques like MiSACE and TES improve the anatomical suitability of endovascular repair and may enhance durability. We report the case of a patient treated with adjunctive advanced endovascular techniques combined with branched repair. The patient provided written consent for publication.

## 2. Case Presentation

**Figure 1:**
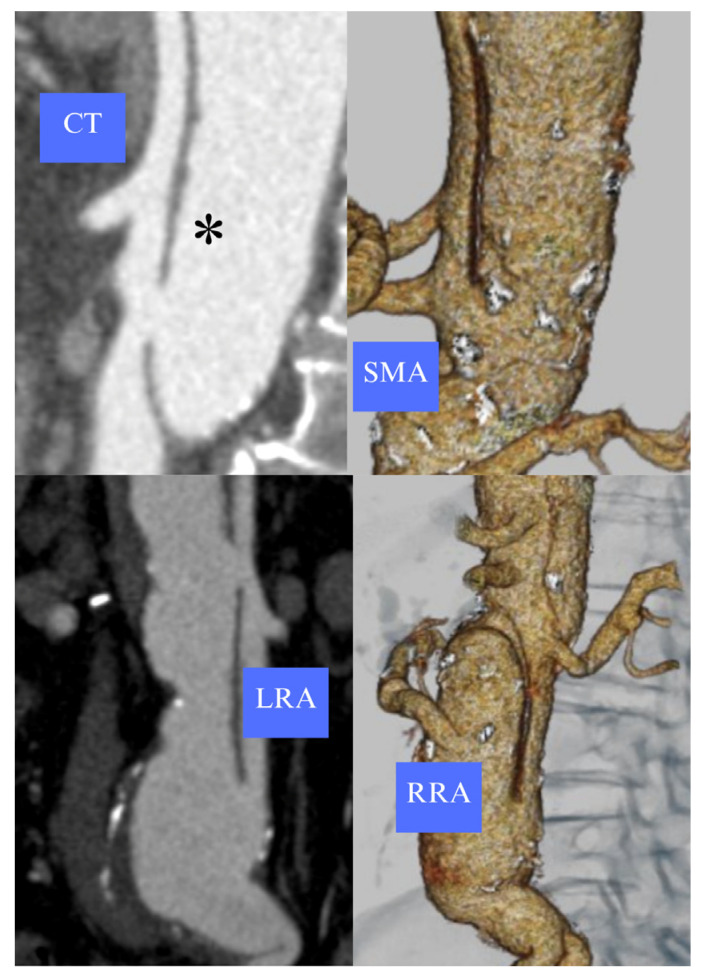
Anatomical features of visceral vessels of a post-dissection type IV TAAA. The dissecting lamella (*) separates the true and false lumen. MPR and 3D reconstructions show an upward-oriented RRA originating from a dilated false lumen, while the CT, SMA, and LRA arise from a narrow true lumen.

A 68-year-old man with chronic obstructive pulmonary disease was managed conservatively for acute TBAD occurring 35 years prior and developed late thoracoabdominal aortic dilatation. He underwent multiple interventions, including open juxta-renal aneurysm repair and a bypass to the right renal artery (2005), open descending thoracic aneurysm repair (2007), and emergent thoracic endovascular aortic repair (TEVAR) for intercostal patch rupture (2014). Recently, the patient suffered acute right limb ischemia caused by a focal dissection of the right external iliac artery, which required cover stenting and intentional sacrifice of the hypogastric artery. Lastly, the patient suffered spontaneous dissection of the left subclavian artery, which subsequently thrombosed. The patient tested negative for CTD genetic screening. Follow-up computed tomography angiography (CTA) showed a 60 mm residual type IV TAAA with celiac trunk (CT), superior mesenteric artery (SMA), and left renal artery (LRA) originating from a narrow true lumen (<15 mm) while the upward-oriented right renal artery (RRA) originated from the false lumen (Figure 1).

**Figure 2:**
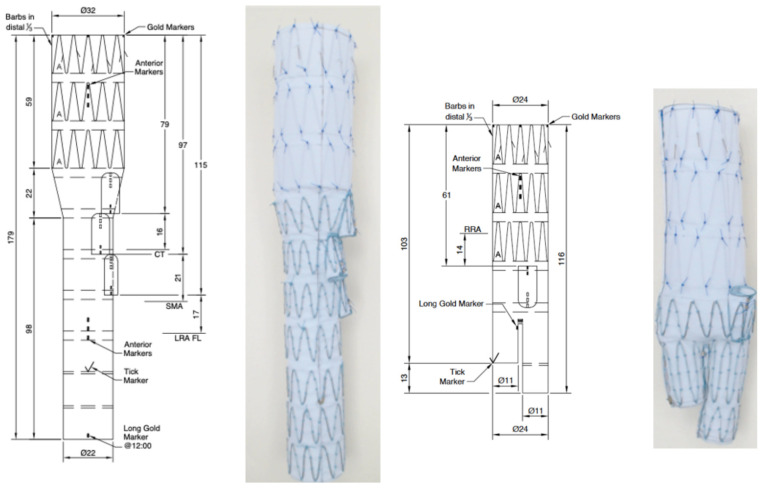
Design and features of the custom-made branch endograft. Two-piece custom-made branched endovascular graft consisting of a thoracic component with three antegrade branches for CT, SMA and LRA, accompanied by a bifurcated abdominal component with a retrograde branch for the upward-oriented RRA.

An endovascular repair was planned with a multifeatured branched custom-made graft with three anterograde branches and one retrograde branch for the RRA (Figure 2, Cook Medical Inc., Brisbane, Australia, and Søborg, Denmark). Due to the impaired spinal cord (SC) collateral network (occluded left subclavian artery, right hypogastric artery, and previous aortic repairs), a staged approach was planned.

Different large intercostal and lumbar arteries (T12-L1-L3) were evident at preoperative CTA, originating from both lumens. Selective coil embolization (Penumbra, Inc. Harbor Bay Parkway, Alameda, CA, USA) was performed two weeks before B-EVAR to precondition the SC while preventing type II endoleak [4,5].

Under systemic heparinization (intravenous bolus of 80–100 U/kg), ultrasound-guided percutaneous catheterization of the common femoral artery was performed using a 6 Fr introducer sheath, 70 cm in length.

Selective catheterization of the intercostal and/or lumbar arteries to be embolized was then obtained using a 0.014″ guidewire and an angiographic microcatheter. The latter was advanced up to the first bifurcation of each segmental artery, where coils were deployed, allowing an uninterrupted collateral network to develop. The procedure was performed under local anesthesia, which allowed spinal cord neurologic assessment throughout the whole process of embolization.

**Figure 3:**
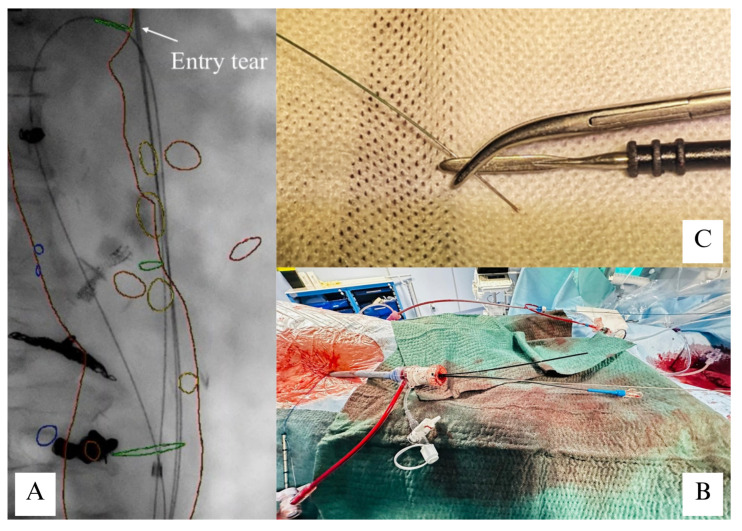
Transcatheter electrified septotomy toolbox. Procedural steps of TES. (**A**) Through-and-through loop creation across the dissecting lamella through the entry tear site. (**B**) Wire exchange over a Navicross catheter within a 20 Fr DrySeal introducer sheath. (**C**) Electrification of the guidewire tip using electrocautery.

After 15 days, TES was performed on the same day as B-EVAR to ease visceral vessel bridging and allow the expansion of the graft [6]. True lumen catheterization occurred through the right femoral access, and intravascular ultrasound (IVUS) (Volcano 0.035′-Philips Healthcare, Amsterdam, The Netherlands) ensured the visualization of the supportive guidewire within the aortic lumen, identifying any accidental deviation from its course. A 20Fr Dryseal introducer sheath (W.L. Gore & Associates, Flagstaff, AZ, USA) was advanced in the true lumen and the IVUS probe was left in place to guide TES. A UF catheter was used to cross the lamella at its proximal edge and catheterize the false lumen with a guidewire. The introducer sheath was double punctured, and the guidewire was snared from the false lumen, building a through-and-through loop (Figure 3A). The guidewire was exchanged over a Navicross catheter (Figure 3B) to a supportive 0.014 guidewire (Asahi Intecc USA Inc., Irvine, CA, USA), which was previously uncoated and angulated in the middle portion. Both ends of the wire were covered with two 4F 0.035 catheters to protect surrounding tissues from thermal injury. Once the distal end was electrified with the electrocautery (Figure 3A), the wire was retracted, splitting the septum.

TES was performed for the total length of the lamella, which extended from the thoracic endograft to the bifurcated abdominal graft already in place and corresponded to the visceral segment of the aorta.

**Figure 4:**
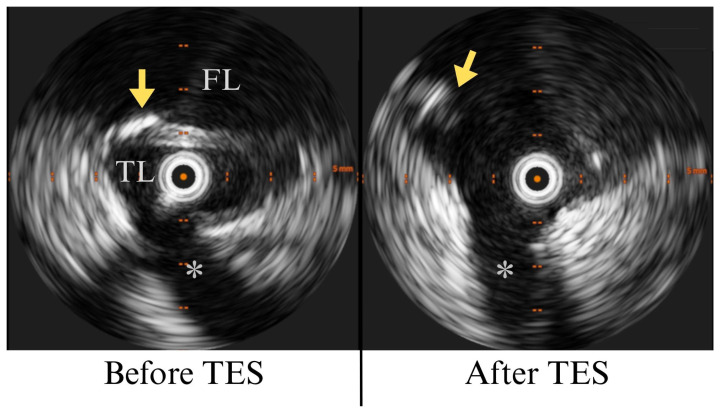
Intraluminal cross-sectional view of the aorta before and after TES. Same-segment intraluminal view of aortic anatomy obtained with IVUS. The true lumen (TL) and false lumen (FL) are separated by a dissection lamella (yellow arrow) near the origin of the LRA (*). TES resulted in complete division of the lamella and the creation of a single aortic lumen.

IVUS documented the behavior of the lamella during the procedure, ensuring it would not obstruct visceral vessel ostia. Blood pressure was carefully monitored during TES, assessing any accidental vessel or aortic perforation, which did not occur. Complete division of the lamella was documented, together with the creation of a single, expanded aortic lumen (Figure 4, Video S1). Selective angiography was obtained for all visceral vessels, ensuring ostial patency after TES. Cannulation of all target vessels was achieved from a transfemoral access, including downward-oriented vessels, which were bridged using a steerable sheath, avoiding upper extremity access [7].

The intervention was performed under general anesthesia due to procedural complexity and duration. No intraoperative SC monitoring tool was implemented but on-table clinical examination immediately after the operation allowed neurological assessment of SC integrity. Prophylactic cerebrospinal fluid drainage (CSFD) and monitoring were not implemented due to the augmented risk of hemorrhagic complications without a clear advantage in terms of SC injury prevention [8]. The optimization of SC vital functions was implemented by maintaining a mean arterial pressure above 80 mmHg and hemoglobin > 100 g/L throughout the operation and hospital stay.

Final angiography and DynaCT demonstrated exclusion of the aneurysm and absence of endoleaks from segmental arteries or stent-graft components.

Blood tests, including complete blood count, renal and hepatic function, and inflammatory status, remained stable throughout the hospitalization. The patient was discharged on postoperative day 4, neurologically intact and without other complications.

**Figure 5:**
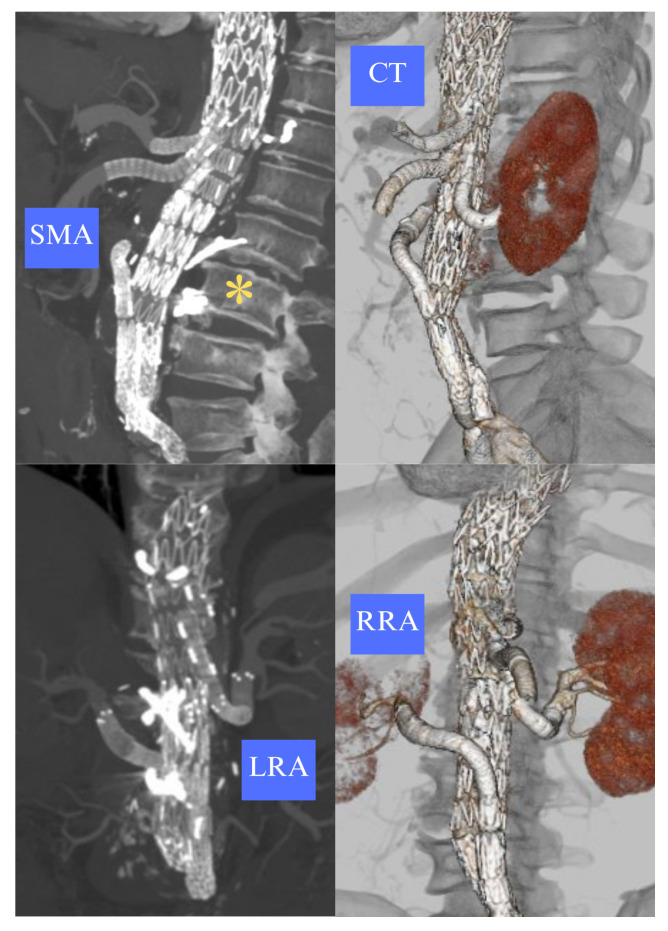
B-EVAR final result. MPR and 3D reconstructions from follow-up CTA showing complete expansion of the custom-made endograft with successful incorporation of CT, SMA, LRA and upward-oriented RRA. Segmental artery coil embolization is visible dorsally to the endograft (*).

An 18-month follow-up CTA confirmed aneurysm exclusion with full expansion of the branched graft and patency of all target vessels (Figure 5). Changes in aneurysm sac volume and diameter were monitored with ARVA, a quantitative imaging platform for aortic volumetric analysis (Incepto Medical, Paris, France), which confirmed stability. No other reinterventions have been performed to this date.

## 3. Discussion

Open surgery has been the standard treatment for PD-TAAAs due to poor endovascular sealing zones and true lumen compression with vessel involvement. Despite these considerations, recent studies suggest that F/B-EVAR is safe in this population, reporting a lower 30-day mortality rate and no difference in overall survival, compared to open surgery [2]. SC ischemia rates are also lower in endovascular repair of PD-TAAAs, maybe due to the major blood loss and absence of staging techniques faced in open surgery. However, the reintervention rate at follow-up, consisting primarily of minor procedures, can reach over 30% at 5 years [9]. Consequently, various adjuncts have been proposed to enhance overall long-term outcomes and reduce reinterventions.

Segmental arteries in PD-TAAAs are often large and arise from both lumens of the dissection. Under these circumstances, standard staging strategies such as proximal stent-grafting or temporary sac perfusion used as SC preconditioning might be ineffective due to persistent back-flow from segmental arteries. In addition, patent large segmental arteries may enhance false lumen perfusion, causing type II endoleak.

Gallitto et al. [5] proposed coil embolization of the entire false lumen to promote thrombosis and prevent endoleaks, while our practice is to perform MiSACE to obtain controlled SC ischemic preconditioning and reduce type II endoleak, minimizing coil artifacts during follow-up CTAs. The possible advantages of this technique have been advocated by different authors, but its safety and effectiveness remain a matter of debate [10]. Moreover, the choice of which intercostal arteries need to be embolized each session, and in which order, still relies on the surgeon’s preference, adding even more variability to the procedure. In our practice, we embolize segmental arteries originating from both lumens, with a diameter greater than 3 mm, especially in the T10-L3 segment, with a minimal interval of five days between different sessions to achieve SC preconditioning [11]. Nevertheless, SC ischemia was never documented as a complication after a session of MiSACE, prior to F/B-EVAR.

Despite technological advancements in F/B-EVAR, its Achilles’ heel remains reinterventions, mostly due to target vessel instability [12]. A narrow true lumen hinders graft expansion, making standard B-EVAR contraindicated in this anatomy. As mentioned above, fenestrated repair is feasible in such cases, but a true lumen diameter of 15 mm would still represent a challenge for endograft expansion. Furthermore, the limited space inside the endograft would complicate the navigability of catheters and introducers for stent deployment. Additionally, the presence of multiple flared stentgrafts inside a small endograft could possibly cause an iatrogenic stenosis of the visceral aorta. Moreover, a diameter mismatch between the true lumen and aneurysm creates a large gap between fenestrations and target arteries, compromising stability and increasing the risk of type III endoleak due to component detachment [13].

Other techniques for fenestrating the lamella in chronic dissections, such as the Knickerbocker, allow thoracic false lumen thrombosis and prevent proximal false lumen endoleaks in the visceral segment when performing F/B-EVAR [14]. We decided not to use the latter technique because we intended to restore the transaortic lumen prior to B-EVAR, not false lumen exclusion. Moreover, in our case, the true lumen was narrow in the visceral segment; therefore, a straight thoracic endograft would not have worked in this situation. TES has been proposed to split the dissecting lamella to facilitate vessel cannulation and to allow liberal use of branched endografts in case of long bridging distances, which are more stable in such anatomy [15]. TES represents a valid solution in case of a chronically compressed true lumen and the absence of entry tears in the lamella at the level of visceral vessel ostia. While TES can be hazardous in acute aortic dissections, reporting high rates of technical failure, due to lamella distal dislodgement and possible vessel coverage or injury [16], in chronic/subacute TBAD, technical success rates are 97–100% [17,18]. Although the procedure might seem highly invasive, harmful complications during TES in chronic aortic dissections have not been reported at the present time. In our report, TES was used to create a single aortic lumen, enabling the full expansion of a branched endograft and facilitating the bridging of target arteries with a standard transfemoral steerable sheath, minimizing the risk of stroke [18,19].

The creation of an electrified loop occurred under IVUS guidance, which ensured the axial visualization of the two lumens and confirmed the presence of both ends of the wire across the lamella, avoiding TES in the wrong location. The synchronized retraction of the IVUS catheter and the electrified loop allowed real-time intraluminal monitoring during TES, providing valuable measurements of aortic diameter and identifying potential geometric modifications in the target vessels. Although the role of IVUS in acute aortic dissection is well-described [20,21,22], its application in chronic dissection remains unclear, highlighting the need of large-cohort studies to define the role of intravascular imaging in aortic surgery.

The implementation of tailored staging strategies (MiSACE) serves not only as a spinal cord preconditioning technique but also as a preventive measure against persistent type II endoleaks. Furthermore, the technical adaptation to complex anatomical challenges, such as B-EVAR with a retrograde branch, and the use of advanced adjuncts like TES and IVUS to manage a narrow true lumen, provide significant novelty to this case. With this approach, we tried to balance immediate procedural success with the long-term durability of the repair.

## 4. Conclusions

Managing PD-TAAAs is particularly demanding due to complex anatomical variations and continuous aortic remodeling. To navigate these challenges effectively, clinicians must possess an extensive repertoire of endovascular skills and bail-out maneuvers. Furthermore, leveraging cutting-edge technologies and advanced techniques, such as MiSACE and TES, is essential for providing viable solutions in the face of highly complex anatomy.

Despite promising results of these rising adjuncts in the treatment of chronic dissections, the absence of complications in one patient does not establish procedural safety and effectiveness, which are yet to be determined in large-cohort prospective studies.

## Data Availability

The original data presented in the study are included in the article, further inquiries can be directed to the corresponding author.

## Supplementary Materials

The following supporting information can be downloaded at: https://www.mdpi.com/article/10.3390/reports9020155/s1. Video S1: Video summary of the entire staged procedure. Legend: Summary of the procedure, including segmental artery coil embolization sessions, IVUS-guided electroseptotomy, and branched endovascular repair, completed with post-procedure cone-beam CT.

## Abbreviations

The following abbreviations are used in this manuscript: Post-dissection thoracoabdominal aortic aneurysms = PD-TAAAs; Fenestrated/branched endovascular aortic repair = F/B-EVAR; Minimally invasive segmental artery coil embolization = MiSACE; Transcatheter electrosurgical septotomy = TES; Type B aortic dissection = TBAD; Connective tissue disorders = CTDs; Thoracic endovascular aortic repair = TEVAR; Computed tomography angiography = CTA; Celiac trunk = CT; Superior mesenteric artery = SMA; Left renal artery = LRA; Right renal artery = RRA; Spinal cord = SC; Intravascular ultrasound = IVUS; Cerebrospinal fluid drainage = CSFD.

## References

[1] Coselli J.S., Lemaire S.A., Preventza O., De La Cruz K.I., Cooley D.A., Price M.D., Stolz A.P., Green S.Y., Arredondo C.N., Rosengart T.K. (2016). Outcomes of 3309 thoracoabdominal aortic aneurysm repairs. J. Thorac. Cardiovasc. Surg..

[2] Cook I.O., Green S.Y., Rebello K.R., Zhang Q., Glover V.A., Zea-Vera R., Moon M.R., LeMaire S.A., Coselli J.S. (2024). Comparison of open thoracoabdominal repair for chronic aortic dissections and aneurysms. J. Vasc. Surg..

[3] Mazzolai L., Teixido-Tura G., Lanzi S., Boc V., Bossone E., Brodmann M., Bura-Rivière A., De Backer J., Deglise S., Della Corte A. (2024). 2024 ESC Guidelines for the management of peripheral arterial and aortic diseases. Eur. Heart J..

[4] Branzan D., Etz C.D., Moche M., Von Aspern K., Staab H., Fuchs J., Bergh F.T., Scheinert D., Schmidt A. (2018). Ischaemic preconditioning of the spinal cord to prevent spinal cord ischaemia during endovascular repair of thoracoabdominal aortic aneurysm: First clinical experience. EuroIntervention.

[5] Gallitto E., Faggioli G., Poliseno C., Cappiello A., Pini R., Vacirca A., Logiacco A., Gargiulo M. (2024). Pre-emptive False Lumen Embolization to Prevent Persistent Type II Endoleak in Fenestrated-Branched Endovascular Repair of Post-Dissection Thoracoabdominal Aortic Aneurysms. J. Endovasc. Ther..

[6] Baghbani A., Savadi S., Mesnard T., Sulzer T., Mirza A.K., Baig S., Timaran C.H., Oderich G.S. (2023). Transcatheter electrosurgical septotomy technique for chronic post-dissection aortic aneurysms. J. Vasc. Surg. Cases Innov. Tech..

[7] Chamseddin K., Timaran C.H., Oderich G.S., Tenorio E.R., Farber M.A., Parodi F.E., Schneider D.B., Schanzer A., Beck A.W., Sweet M.P. (2023). Comparison of upper extremity and transfemoral access for fenestrated-branched endovascular aortic repair. J. Vasc. Surg..

[8] Leone N., D’oria M., Mani K., Oderich G., Maleti G., Bartolotti L.A., Silingardi R., Lepidi S., Gennai S. (2024). Systematic review and meta-analysis of cerebrospinal fluid drain-related mortality and morbidity after fenestrated-branched endovascular aortic repair. J. Vasc. Surg..

[9] Gallitto E., Faggioli G., Melissano G., Fargion A., Isernia G., Bertoglio L., Simonte G., Lenti M., Pratesi C., Chiesa R. (2022). Fenestrated and Branched Endografts for Post-Dissection Thoraco-Abdominal Aneurysms: Results of a National Multicentre Study and Literature Review. Eur. J. Vasc. Endovasc. Surg..

[10] Addas J.A.K., Mafeld S., Mahmood D.N., Sidhu A., Ouzounian M., Lindsay T.F., Tan K.T. (2022). Minimally Invasive Segmental Artery Coil Embolization (MISACE) Prior to Endovascular Thoracoabdominal Aortic Aneurysm Repair. Cardiovasc. Interv. Radiol..

[11] Etz C.D., Kari F.A., Mueller C.S., Brenner R.M., Lin H.-M., Griepp R.B. (2011). The collateral network concept: Remodeling of the arterial collateral network after experimental segmental artery sacrifice. J. Thorac. Cardiovasc. Surg..

[12] Abdelhalim M.A., Tenorio E.R., Oderich G.S., Haulon S., Warren G., Adam D., Claridge M., Butt T., Abisi S., Dias N.V. (2023). From the Society for Vascular Surgery Multicenter trans-Atlantic experience with fenestrated-branched endovascular aortic repair of chronic post-dissection thoracoabdominal aortic aneurysms. J. Vasc. Surg..

[13] Chait J., Tenorio E.R., Mendes B.C., Lima G.B.B., Marcondes G.B., Wong J., Macedo T.A., De Martino R.R., Oderich G.S. (2022). Impact of gap distance between fenestration and aortic wall on target artery instability following fenestrated-branched endovascular aortic repair. J. Vasc. Surg..

[14] Rohlffs F., Tsilimparis N., Panuccio G., Heidemann F., Behrendt C.-A., Kölbel T. (2023). The Knickerbocker Technique: Technical Aspects and Single-Center Results of a New Endovascular Method for False Lumen Occlusion in Chronic Aortic Dissection. J. Endovasc. Ther..

[15] Squizzato F., Antonello M., Forcella E., Coppadoro S., Colacchio C., Xodo A., Grego F., Piazza M. (2022). Geometrical determinants of target vessel instability in fenestrated endovascular aortic repair. J. Vasc. Surg..

[16] Ruiter Kanamori L., Tenorio E.R., Babocs D., Savadi S., Baghbani-Oskouei A., Huang Y., Figueroa A., Tanenbaum M., Filho J.E.C., Baig M. (2024). Indications, safety, and effectiveness of transcatheter electrosurgical septotomy during endovascular repair of aortic dissections. J. Vasc. Surg..

[17] Tanenbaum M.T., Figueroa A.V., Lee K.B., Filho J.E.C., Gonzalez M.S., Baig M.S., Timaran C.H. (2024). Early results of transcatheter electrosurgical aortic septotomy for endovascular repair of chronic dissecting aortoiliac aneurysms. J. Vasc. Surg. Cases Innov. Tech..

[18] Mario D., Alessandro G., Giovanni P., Gianbattista P., Rocco G., Mauro G., Nicola M., Roberto C., Sandro L., Luca B. (2023). Total Transfemoral Branched Endovascular Thoracoabdominal Aortic Repair (TORCH2): Short-term and 1-Year Outcomes from a National Multicenter Registry. J. Endovasc. Ther..

[19] Melloni A., Bertoglio L., Eynde W.V.D., Agrusa C.J., Parlani G., Howard D.P.J., Rio J., Fazzini S., Mansour W., Dias N.V. (2025). Outcomes of Percutaneous Access to the First Versus Third Segment of Axillary Artery During Aortic Procedures. J. Endovasc. Ther..

[20] Belkin N., Jackson B.M., Foley P.J., Damrauer S.M., Kalapatapu V., Golden M.A., Fairman R.M., Wang G.J. (2020). The use of intravascular ultrasound in the treatment of type B aortic dissection with thoracic endovascular aneurysm repair is associated with improved long-term survival. J. Vasc. Surg..

[21] Koschyk D.H., Nienaber C.A., Knap M., Hofmann T., Kodolitsch Y.V., Skriabina V., Ismail M., Franzen O., Rehders T.C., Dieckmann C. (2005). How to guide stent-graft implantation in type B aortic dissection? Comparison of angiography, transesophageal echocardiography, and intravascular ultrasound. Circulation.

[22] Kyriakou A., Ibrahim A., Oberhuber A. (2024). Intravascular Ultrasound Enhances the PETTICOAT Technique in Endovascular Therapy for Complicated Type B Aortic Dissection with Malperfusion Syndrome. Ann. Vasc. Surg..

